# HIV incidence among men who have sex with men in mainland China: a systematic review protocol

**DOI:** 10.1186/s13643-021-01828-w

**Published:** 2021-10-27

**Authors:** Wenting Huang, Liming Wang, Mi Guodong, Ryan J. Zahn, Jennifer Taussig, Shenita R. Peterson, Stefan Baral, Renee H. Moore, Xiaojie Huang, Jianhua Hou, Patrick S. Sullivan, Aaron J. Siegler

**Affiliations:** 1 Department of Behavioral Sciences and Health Education, Emory University, Atlanta, GA, 1518 Clifton Road, Atlanta, GA 30322 USA; 2 Beijing Danlan Media Limited, Beijing, China; 3 Department of Epidemiology, Rollins School of Public Health, Emory University, Atlanta, USA; 4 Woodruff Health Sciences Center Library, Emory University, Atlanta, USA; 5 Department of Epidemiology, John Hopkins School of Public Health, Baltimore, USA; 6 Biostatistics and Bioinformatics, Emory University, Atlanta, USA; 7 Beijing YouAn Hospital, Capital Medical University, Beijing, China

**Keywords:** HIV, Incidence, Epidemiology, MSM, China

## Abstract

**Background:**

Men who have sex with men (MSM) are disproportionately affected by HIV in China. Globally, younger MSM are at higher risk for incident HIV infections, but there has been substantial variation in the estimates of age-stratified HIV incidence among MSM in mainland China, potentially due to regional differences in the nature of the epidemic. Given the need for quality epidemiological data to meet the global goal of ending new HIV infections by 2030, this systematic review and meta-analysis aims to determine age-stratified HIV incidence in mainland China, including consideration by geographic region and time.

**Methods:**

This review will include longitudinal studies, cross-sectional surveys, and surveillance reports among MSM in mainland China that have reported HIV incidence. We will search studies and reports published from January 1, 2003, to April 30, 2020, in both English and Chinese language literature databases. For each study considered, two reviewers will independently screen, determine eligibility, and extract relevant data, with discrepancies resolved by consensus of a third reviewer. The methodological quality of included studies will be assessed by the Quality Assessment Tool for Systematic Reviews of Observational Studies Score (QATSO). We will develop age-stratified estimates of HIV incidence with geographic variations and temporal trends. Heterogeneity will be examined using statistical techniques appropriate to the dataset. For subgroup analyses, we will conduct mixed-effects meta-analysis models.

**Discussion:**

This review will contribute to a better understanding of the HIV epidemic among MSM in mainland China by providing age-stratified estimates of HIV incidence with a portrayal of geographic and temporal variations. Findings will reflect epidemic dynamics, informing local and national intervention programs and policies for HIV prevention, and providing estimation data to inform future research among MSM in China.

**Systematic review registration:**

PROSPERO ID 154834

## Introduction

Despite the low HIV prevalence among the general population in China, men who have sex with men (MSM) are disproportionately affected by HIV [1]. The nationwide sentinel surveillance system estimated that the HIV prevalence among MSM has increased from 1% in 2003 to 8% in 2014; MSM were the only at-risk population with increased HIV prevalence during this period [2].

In a number of settings, younger MSM are at increased risk for HIV infection [3–10]. Countries in North American, Western Europe, and Australia have reported an increasing proportion of HIV diagnoses among young MSM [3]. Young MSM were also reported to have less access to HIV prevention and treatment services than older MSM in both high- and low-income counties [7–10]. In China, the number of HIV infections captured by the sentinel surveillance system has increased among young MSM during 2005‑2012 [11]. Similarly, young age group was associated with higher HIV prevalence among MSM in several longitudinal studies and meta-analyses [12–14]. A recent study found that young MSM had both a higher HIV prevalence (5.4% vs. 3.6%) [5]. Conversely, several studies have found older MSM age groups had higher HIV prevalence [15, 16]. A recent systematic analysis of 355 cross-sectional studies in China indicated that MSM aged 50 years and older had the highest HIV prevalence of 19.3%, compared with the overall national prevalence of 5.7% [16], indicating that HIV incidence may not plateau but instead continue at high levels throughout adulthood. Together, these empirical findings suggest that age is a critical component in understanding the epidemic of HIV among MSM that merits further exploration.

In addition to age variation, several meta-analyses assessing HIV prevalence indicate considerable regional variation of the HIV epidemic among MSM in mainland China [11, 16, 17]. In general, the southwest has been observed to have the highest HIV prevalence, followed by the southeastern coastal regions and northeast regions [11, 16, 17]. Differences in the distribution of HIV prevalence may vary across regions and by age groups within regions; in a country with over 1 billion persons, it is important to consider regional differences by such essential covariates. Similarly, temporal trends may differ by region. For instance, a repeated cross-sectional study in Beijing showed a rapid doubling of HIV prevalence among MSM (baseline 5%, follow-up 10%) between 2008 and 2011 [18]. This is in contrast to the sentinel surveillance data in Chengdu, southwest China, which showed a more stable, high HIV prevalence between 2009 and 2012 (baseline 14%, follow-up 16%) [19]. These variations in the HIV prevalence across studies indicate the need for a systematic exploration of HIV epidemic dynamic, taking into consideration geographic variation and temporal trends among MSM in mainland China. The HIV prevalence between 2001 and 2018 among Chinese MSM has been recently summarized, finding national HIV prevalence among MSM was estimated to be 5.7% (5.4‑6.1%), with variation by geographic location, age, and other sociodemographic and behavioral factors including education, partners seeking, and condom use [16].

There remains a need to characterize HIV incidence among MSM in China, with the most recent review conducted over a decade ago, estimating incidence based on three cohort studies and nine cross-sectional studies [20]. To optimally inform policy and interventions, there is a need to review and synthesize HIV incidence among Chinese MSM to aggregate data regarding this key epidemic parameter, including assessment of variation by age groups, regions, and temporal change.

### Objectives

Our study aims to provide comprehensive and updated information on HIV incidence among Chinese MSM, with estimates stratification by age and temporal trends at national and regional levels. This information may inform policy decisions and resource allocations regarding HIV prevention services.

Review questions:

1. What is the age-stratified HIV incidence among MSM residing in mainland China?
2. What are the regional differences in and temporal trends of HIV incidence among MSM residing in mainland China?
3. What factors are associated with temporal changes in HIV incidence in MSM residing in mainland China?

## Methods

### Design and reporting

This review will adopt the guidelines recommended by the Center for Reviews and Dissemination [21], with data reporting and recording following the guidelines of The Preferred Reporting Items for Systematic Reviews and Meta-Analyses (PRISMA) [22]. Protocol development followed the principles of the updated PRISMA statement for Protocols (PRISMA-P) [23, 24]. The protocol has been submitted for registration in the PROSPERO International Prospective Register of systematic reviews, with the ID number of 154834.

### Criteria for considering studies for this review

#### Inclusion criteria

- *Study types*: Observational studies including longitudinal study, cross-sectional surveys, and surveillance reports
- *Study sample size*: ≥ 50
- *Study populations*: MSM and/or transgender women residing in mainland China, not including Taiwan, Hong Kong, and Macau
- *Study outcomes*: Laboratory determined HIV incidence figure.
- *Quality criteria*: Published in a peer-reviewed journal, presented as an abstract at a scientific conference, or available on the web from governmental or non-governmental sources. If the study/report was presented at a conference, it needs to be presented during the period between the dates of April 30, 2018, and April 30, 2020, and be presented at selected conferences. We use this short date range because we expect less recent findings should be published in peer-reviewed journals.
- *Study data collection timeframe*: On or after January 1, 2003
- *Publication timeframe*: January 1, 2003, to April 30, 2020
- *Publication language*: English or Chinese

#### Exclusion criteria

1. Study is not presenting new data.
2. Study sample size < 50.
3. Study does not have a laboratory-confirmed HIV incidence result.
4. Study is not observational, meaning it is not a longitudinal study, a cross-sectional survey, or a surveillance report.
5. Study is not among Chinese MSM and/or transgender women who reside in mainland China, including but not limited to those residing in Taiwan, Hong Kong, and Macau.
6. Study data collection period occurs prior to January 1, 2003.
7. Study publication time frame is not between January 1, 2003, and April 30, 2020.
8. Study publication is not published in English or Chinese.
9. Study is neither published in a peer-reviewed journal, available on the web from governmental or non-governmental sources, nor presented at selected conferences during April 30, 2018, to April 30, 2020.

The timeframe for study data collection is specified in addition to publication timeframe because this study aims to assess the HIV incidence among MSM in the past 15 years; we extended study eligibility two additional years (from 2003 forward) to include studies that report longitudinal data that may overlap into the 15-year period of interest. We believe this period will provide sufficient evidence for present program and research planning. We excluded RCT because such trials involve interventions, usually specifically intended to reduce HIV incidence. Even among control participants, standard of care intervention such as referrals to care and distribution of condoms and condom-compatible lubricant often may influence HIV incidence.

### Search strategy for identifying relevant studies

#### Information sources

In partnership with information management specialists, a comprehensive Medline search on HIV incidence among Chinese MSM from January 1, 2003, to April 30, 2020, will be conducted in the following databases: (1) for English publications: PubMed, CENTRAL, and Web of Science; (2) for Chinese publications: China Academic Journals Full-text Database (CJFD)/China National Knowledge Infrastructure (CNKI), China Science and Technology Journal Database (VIP), and WanFang Data. Conference abstracts will be searched from the online archives of the International AIDS Conference (IAC) and the CROI Conference for English publications, as well as the National Conference of HIV/AIDS for Chinese publications.

Other data sources will include Chinese national surveillance system data reports and project reports or documents developed by large international and local non-governmental organizations that provide current or previous support for HIV-related programs in China (if available). In addition, we will review the citation list of the included articles to identify additional studies and reports.

#### Search strategy

In this review, we will have five search queries connected by “AND”: (1) the search query for HIV will be [“HIV” OR or “human immunodeficiency virus” OR “AIDS” OR “Acquired Immune Deficiency Syndrome” OR “HIV/AIDS” OR “seropositive”]; (2) the search query for incidence will be [“incidence” OR “infection rate” OR “infection rates” OR “new cases”]; (3) the search query for study population will be [“men who have sex with men” OR “men having sex with men” OR “men who had sex with men” OR “MSM” OR “homosex” OR “gay” OR “gays” OR “queer” OR “bisex” OR “transgender” OR “transsex” OR “trans”]; (4) the search query for study geographic location will be [“China” OR “Chinese”]; and (5) the language and publication date query will be [(Chinese[lang] OR English[lang]) AND (“2003/01/01”[PDAT]: “2019/08/31”[PDAT])].

The Chinese search terms will be translated from English and adapted based on the terminology used in Chinese publications.

#### Review strategy

A review team of at least four reviewers will review the titles and abstracts identified from the initial search (at least two reviewers fluent in each language). Two independent reviewers will use the inclusion and exclusion criteria to assign each identified publication into one of three categories: (1) Immediate inclusion: when the article appears to meet the inclusion criteria for the review; (2) Pull to check: when the article may or may not meet the inclusion criteria, and the full text of the article must be reviewed before a final decision about inclusion can be made; (3) Immediate exclusion: when the article clearly does not meet the inclusion criteria for the review and no further consideration is necessary. Review results will be entered into using Covidence, a software for managing and streamlining systematic reviews. After the initial screening, full articles will be obtained for all abstracts selected in the groups of “immediate inclusion” and “pull to check.” Two reviewers will review each full-text article independently, making a final determination regarding study inclusion. A screening guide will be used to ensure the consistency of selection criteria by all reviewers. Disagreement between the independent reviewers on final inclusion will be resolved through consensus or referral to a third reviewer. In addition, a bilingual reviewer will facilitate the unified implementation of reviewing procedures for both English- and Chinese-language abstracts and full-text articles. Finally, reviewers will review the citation list of included articles to identify additional studies.

### Data abstraction and management

For each included article and report, data will be extracted independently by two reviewers using a standardized data extraction form. Differences in data extraction will be resolved through consensus and referral to a senior reviewer when necessary.

Information to be abstracted include the following:

1. Study identification: the name of the first author, the type of citation, and the year of publication
2. Study description: geographic location (e.g., the name of the region/province/city), study design (e.g., longitudinal study, cross-sectional); data collection time frame, design of data collection (prospective vs. retrospective), loss to follow-up
3. Participant description: recruitment, eligibility criteria, age, sex, gender, and additional available demographic characteristics such as education or income
4. Outcomes: HIV incidence by year. The information that will be extracted includes the laboratory methods for HIV diagnosis (e.g., antibody test, BED, avidity), and the primary data (sample size and the number of BED or avidity positive). These primary data will then be used to calculate the estimated incidence rate.
5. Behavioral covariates: Available behavioral risk factors will also be extracted, such as the number of sexual partners, the number of sex with casual male partners, the number of syphilis infections, and the number of consistent condom use.
6. For multinational studies including China, only data relevant to China will be extracted. For studies including multiple geographic locations within China, data (when available) will be extracted separately for each location.
7. Risk of bias assessment: Quality Assessment Tool for Systematic Reviews of Observational Studies Score (QATSO)

### Appraisal of methodological quality and risk of bias

Methodological quality of included studies will be assessed using a modified checklist based on the Quality Assessment Tool for Systematic Reviews of Observational Studies Score (QATSO) [25, 26]. QATSO as implemented in this study will consist of five items to assess potential bias introduced by study designs: including external validity (1 item), reporting (2 items), bias (1 item), and confounding factors (1 item). We will largely use QATSO unaltered, but for some items will adapt it to be appropriate for this HIV incidence review. For instance, we will use longitudinal response rates instead of cross-sectional response rates for item reporting. The QATSO tool will allow for studies to be classified as either good, satisfactory, and poor quality. We will report publication bias using a statistical test such as Egger’s test. The *p* value of the Egger intercept test less than 0.05 will be considered as indicating statistical significance.

### Data analyses, synthesis, and heterogeneity assessment

Data will be analyzed according to coding categories and outcomes. The extracted data will be used to generate age-stratified estimates of HIV incidence, with geographic distribution and temporal trends of HIV incidence among MSM in mainland China.

Review question one will be assessed with a forest plot including the ways studies are pooled and the estimates for each pooled group with confidence intervals. For review question two, we will conduct mixed-effects meta-regression models to report the estimates for incidence by year or larger time frame between 2002 and 2020 with confidence intervals, and perform similar analyses by geographic region. Mixed-effects model is selected to optimize our longitudinal analysis, allowing for flexibility in selection of time periods for aggregation. For review question three, we will develop a joinpoint model or piecewise regression model to explore the influence of various factors on changes in HIV incidence. Factors considered for this analysis include demographics, region, and recruitment source. Our final modeling strategy will be based on data availability and model fit.

Data will be analyzed using STATA (Stata Crop Version 16.0, Texas, USA). For longitudinal studies, the estimate of the HIV incidence rate among MSM will be calculated by the number of detected HIV infections divided by person-years. For assay-based incidence assessments, estimates of incidence will be measured based on the positive testing results from BED or avidity tests as having a recent infection. If available, we will standardize assay-based incidence assessments to a person-years measure for comparability to cohort-determined incidence. We will seek to exclude patients already on antiviral therapy if possible. Included studies will be pooled for meta-analysis as appropriate, but we will also examine the observed incidence and assay-based incidence estimates separately. Heterogeneity will be examined using the Chi-squared test on Cochrane’s *Q* statistic based on *I*-squared values [27]. The heterogeneity will be defined as a *p* value of the *Q* test less than 0.005 or an *I*-squared value above 50%. If substantial heterogeneity is identified, we will use a fixed-effects model to detect potential sources of heterogeneity.

We will assess the HIV incidence among the following subgroups: transgender women, age, geographic location, and years of studies being conducted. We will also assess these subgroups of MSM depending on data availability: gay MSM, bisexual MSM, sex worker, and general MSM. If studies are significantly different by study population or geographic location, a narrative will be developed to summarize differences. We will also consider the appropriateness of creating a pooled estimate.

## Discussion

Our review seeks to provide a summary of age-stratified, regional, and temporal variation of HIV incidence among MSM in mainland China. We anticipate several uses for such data: (1) informing local strategy for HIV prevention by providing geographic region data, (2) informing national programs and policies for HIV prevention through a better understanding of the variation of HIV incidence regarding age, geographic region, and time, among MSM, and (3) providing estimation data for future research. The estimated HIV incidence will also be used in mathematical modeling for the potential impact of scaling-up different combinations of HIV prevention packages for Chinese MSM. The findings of this systematic review will be published in a peer-review journal and will be presented to relevant health authorities to inform HIV prevention and intervention strategies.

This study will have a number of limitations. One challenge we anticipate is addressing instances of multiple publications from the same dataset. To address this, we will select the publication with the most person-years of observation and/or that reports study outcomes disaggregated by variables of interest such as region. Another challenge will be identifying overlap of articles published in both English and Chinese. To address this, we will employ bilingual reviewers to identify articles published in both languages. When an article is found to be published in both languages, we will delete one to avoid duplication.

## Data Availability

The datasets used and/or analyzed during the current study are available from the corresponding author on reasonable request.

## Abbreviations

AIDS: Acquired immune deficiency syndrome
ART: Antiretroviral therapy
CI: Confidence interval
CROI: Conference on retroviruses and opportunistic infections
HIV: Human immunodeficiency virus
IAC: International AIDS Conference
MSM: Men who have sex with men
PLWH: People living with HIV
PRISMA: Systematic Reviews and Meta-Analyses
PRISMA-P: Systematic Reviews and Meta-Analyses statement for Protocols
RCT: Randomized controlled trials
WHO: World Health Organization
